# ADFCNN-BiLSTM: A Deep Neural Network Based on Attention and Deformable Convolution for Network Intrusion Detection

**DOI:** 10.3390/s25051382

**Published:** 2025-02-24

**Authors:** Bin Li, Jie Li, Mingyu Jia

**Affiliations:** School of Computer Science, Northeast Electric Power University, Jilin 132012, China

**Keywords:** network intrusion detection, bidirectional long short-term memory, deformable convolution, attention mechanism

## Abstract

Network intrusion detection systems can identify intrusion behavior in a network by analyzing network traffic data. It is challenging to detect a very small proportion of intrusion data from massive network traffic and identify the attack class in intrusion detection tasks. Many existing intrusion detection studies often fail to fully extract the spatial features of network traffic and make reasonable use of temporal features. In this paper, we propose ADFCNN-BiLSTM, a novel deep neural network for network intrusion detection. ADFCNN-BiLSTM uses deformable convolution and an attention mechanism to adaptively extract the spatial features of network traffic data, and it pays attention to the important features from both channel and spatial perspectives. It uses BiLSTM to mine the temporal features from the traffic data and employs the multi-head attention mechanism to allow the network to focus on the time-series information related to suspicious traffic. In addition, ADFCNN-BiLSTM addresses the issue of class imbalance during the training process at both the data level and algorithm level. We evaluated the proposed ADFCNN-BiLSTM on three standard datasets, i.e., NSL-KDD, UNSW-NB15, and CICDDoS2019. The experimental results show that ADFCNN-BiLSTM outperforms the state-of-the-art model in terms of accuracy, detection rate, and false-positive rate.

## 1. Introduction

A network intrusion detection system (NIDS) can identify potential attacks and threats [1] by analyzing network packets, thereby protecting the security of private information or network systems. The rapid proliferation of the IoT [2] and information technology (IT) infrastructure and services in businesses and enterprises has created opportunities for new types of cyberattacks [3]. As one of the effective methods to combat various network threats, NIDSs have become a popular research direction in the field of network security.

Traditional network intrusion detection systems are divided into signature-based NIDSs and anomaly-based NIDSs [4]. Signature-based NIDSs adopt a blacklist mechanism. These methods detect anomalies by comparing the features of network traffic with historical attack samples. However, signature-based NIDSs can only detect specific attack types [5]. Anomaly-based NIDSs use a whitelist mechanism. These methods establish a normal behavior model, and other behaviors that fall outside the model are classified as abnormal. As a result, these methods may have a high false-positive rate [6].

Deep learning (DL) has powerful pattern recognition and automatic feature extraction abilities. DL-based NIDSs have gradually replaced traditional NIDSs [7,8]. DL-based NIDSs often use CNNs [9], RNNs [10], LSTM [11], and BiLSTM [12] to complete the task of network intrusion detection. A CNN can effectively extract local features, but it struggles to learn temporal patterns. An RNN can capture both long-term and short-term dependencies, but there are the issues of gradient vanishing and exploding. LSTM solves the issue of gradient vanishing through a gate mechanism, but it can only learn sequence features from one direction. Compared with LSTM, BiLSTM can consider the impact of forward and backward information on the current sample, reducing the false-positive rate of detection. However, BiLSTM only extracts temporal features and ignores spatial features. Therefore, current mainstream research has combined BiLSTM with a CNN [13], using the CNN to extract spatial features and BiLSTM to extract time-series features so as to more accurately identify complex attack patterns.

Although the CNN-BiLSTM network has performed excellently in NIDS tasks, there are still some problems: 1. The network makes it difficult to identify minority attacks, resulting in a high false-positive rate [14]. 2. The network cannot sufficiently extract the spatial features of network traffic. 3. The network fails to make rational use of temporal features. In order to solve the above problems, we propose an intrusion detection network based on attention and deformable convolution (ADFCNN-BiLSTM). The contributions of this paper are as follows:

(1) We combine the data-level and algorithm-level methods to alleviate class imbalance issues. At the data level, we oversample the minority samples and undersample the majority samples to balance the sample distribution. At the algorithm level, we alleviate the impact of imbalance on network training by increasing the network’s attention to minority class data during the training process.
(2) We propose a spatial feature extraction module based on deformable convolution and attention. The module can adaptively adjust the receptive field when extracting traffic data features and capture important spatial features at the same time.
(3) We propose a temporal feature extraction module based on a multi-head attention mechanism, which enables the network to focus on the temporal information corresponding to suspicious traffic.
(4) We evaluated the proposed ADFCNN-BiLSTM using the NSL-KDD, UNSW-NB15, and CICDDoS2019 datasets. The ablation experiment proved the rationality of the ADFCNN-BiLSTM network.

The rest of this paper is organized as follows: Section 2 discusses related work. Section 3 provides a detailed introduction to the proposed ADFCNN-BiLSTM. The experimental analysis is presented in Section 4. Section 5 concludes this paper.

## 2. Related Work

### 2.1. Imbalanced Data-Processing Methods

To address the imbalance issues of network traffic samples, researchers have proposed various methods. These methods are divided into data-level and algorithm-level methods according to their processing mechanisms [15].

The data-level method aims to balance the data by adjusting the distribution of training samples. It mainly includes oversampling, undersampling, and data generation methods [16,17,18]. Li et al. [19] attempted to address the issue of data imbalance by using VAE-WGAN to generate data. However, this method requires high-quality traffic data, which can easily lead to network instability. In contrast, sampling techniques such as SMOTE [20] and ENN [21] have received extensive attention due to their high robustness and low complexity. Uallh et al. [22] used SMOTE to balance abnormal traffic so that the network could detect minority attacks. Sayem et al. [23] applied the SMOTE and ENN technologies to decrease the false-positive rate related to unbalanced data. Shin et al. [24] integrated SMOTE and ENN into a categorical framework. However, relying solely on the oversampling method cannot fundamentally address the underlying problems caused by data imbalance, such as the internal imbalance between different samples, the nonlinear distribution of the feature space, and so on.

Algorithm-level methods, such as ensemble learning [25], the reweighting method [26], and cost-sensitive learning [27], are used to deal with the challenges caused by sample imbalance by modifying the objective function or training strategy of the learning algorithm. These methods make the model less susceptible to data fluctuations and noise. Zhou et al. [27] used the M-AdaBoost-A algorithm to incorporate the area under the curve into the enhancement process to address the class imbalance in network intrusion detection. Xu et al. [28] used focus loss to address the problem of class imbalance. Dai et al. [29] applied EQLv2 as the loss function to pay more attention to minority samples during model training, thereby avoiding the insufficient learning of minority samples due to their scarcity. Compared with other algorithm-level methods, the EQLv2 loss function provides a more concise and effective way to handle data imbalance, avoiding the additional complexity introduced by the ensemble learning and reweighting methods. However, since EQLv2 usually does not change the distribution of training samples, it may not be able to gain significant improvement when dealing with scarce samples.

### 2.2. NIDS Based on CNN-BiLSTM

Jay et al. [13] proposed a deep learning network combining a CNN and BiLSTM, which can extract spatiotemporal features from traffic data. CNN-BiLSTM combines the advantages of a CNN in spatial feature extraction and BiLSTM in sequence feature extraction. CNN-BiLSTM has become the mainstream network framework for network intrusion detection tasks [28]. However, some more novel deep learning frameworks, such as transformers, have been applied to network intrusion detection tasks. The current mainstream research still uses the CNN-BiLSTM framework to complete network intrusion detection tasks. For example, Zhang et al. [30] proposed a CNN-based sparse transformer intrusion detection model that extracts temporal features through a sparse transformer. This method reduces the parameters of the model but weakens its performance. Gao et al. [29] used the CNN-BiLSTM framework to extract spatiotemporal features and optimized the network with C5.0 Decision. Jouhari et al. [31] designed a NIDS for resource-constrained IoT devices based on lightweight CNN-BiLSTM. This method reduces the computational resources while ensuring the detection performance. Said et al. [32] used a random forest classifier and recursive feature elimination method to select features. The selected features were then sent to the hybrid CNN and BiLSTM network to enhance both its binary and multi-classification performance in network intrusion detection. The experimental results show that the hybrid network not only achieves high accuracy but also effectively reduces the training time. All the above methods use CNNs to extract the features of network traffic. However, since the receptive field of a CNN is fixed, it cannot be flexibly adjusted according to the traffic data. These methods also lack the ability to focus on the relevant traffic features at important moments.

## 3. Methodology

### 3.1. ADFCNN-BiLSTM Network

**Figure 1:**
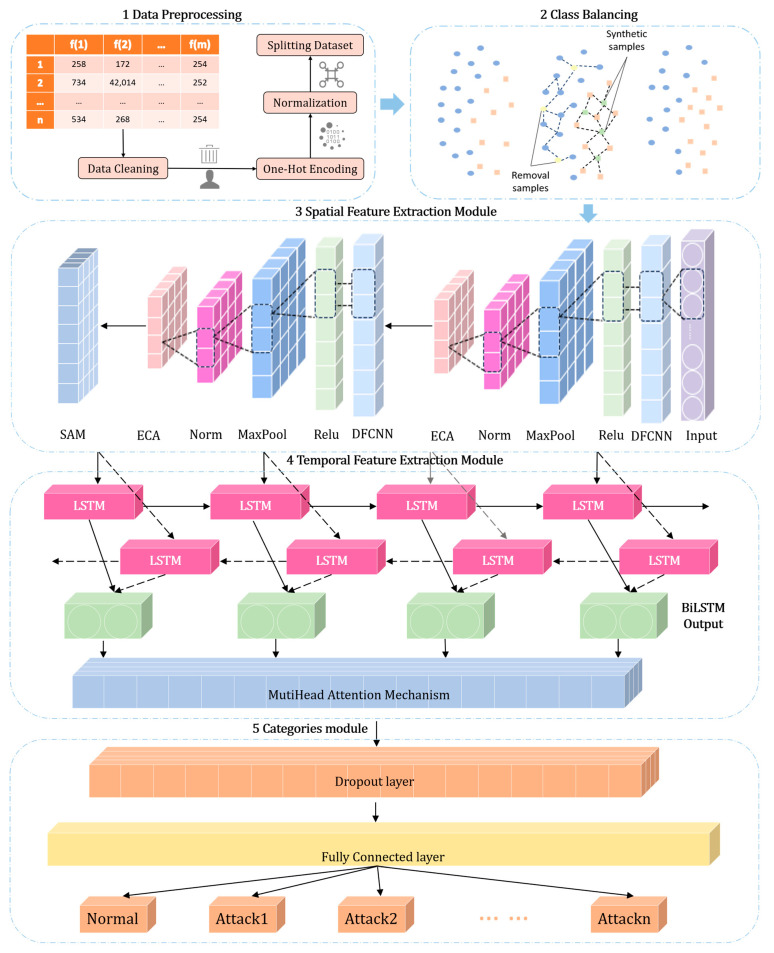
Network architecture of ADFCNN-BiLSTM.

Figure 1 shows the architecture of the ADFCNN-BiLSTM network. The ADFCNN-BiLSTM is composed of three modules: the spatial feature extraction module based on deformable convolution (DFCNN) and attention, the temporal feature extraction module based on multi-head attention (MHA), and the categories module. Before the network traffic data enters the ADFCNN-BiLSTM network, it needs to undergo two steps: data preprocessing and class balancing.

Data preprocessing (step 1 in Figure 1) transforms the original network traffic into a form that can be processed by the deep learning network. For example, it can remove redundant data, correct errors, use one-hot encoding to convert discrete non-numeric features into numeric features, and standardize the data to the [0, 1] interval through max–min normalization.

Class balancing (step 2 in Figure 1) is performed on the traffic data after data preprocessing. The minority samples are expanded using the synthetic minority oversampling technique (SMOTE), and the majority samples are undersampled by the edit nearest neighbor (ENN) technique in order to optimize the data distribution and provide a more balanced dataset for model training.

The resampled data are sent to the spatial feature extraction module (step 3 in Figure 1) to extract spatial features. In this module, the DFCNN adaptively adjusts the receptive field according to the data flow. The module also uses efficient channel attention (ECA) to selectively focus on important channel features. Additionally, based on ECA, the spatial attention mechanism (SAM) is used to locate the important area in the input space. The combination of the DFCNN, ECA, and SAM enables the module to focus on key feature areas and reduce redundant information, thereby enhancing the network’s robustness and expressive ability in complex scenarios.

The features extracted by the spatial feature extraction module are concatenated along the channel dimension and then input into the temporal feature extraction module (step 4 in Figure 1) to extract temporal features. The temporal feature extraction module is based on the BiLSTM network framework, with multi-head attention (MHA) added behind it. MHA helps the network focus on important time points in the time series, which is more beneficial for identifying intrusion behavior in the network.

In the categories module (step 5 in Figure 1), the dropout layer is used to suppress feature redundancy and reduce the risk of overfitting. The extracted spatiotemporal features are fused using the fully connected layer to generate high-order feature representations. Finally, the high-dimensional features are mapped onto the specific classification through the fully connected layer.

### 3.2. Spatial Feature Extraction Module Based on DFCNN and Attention

**Figure 2:**
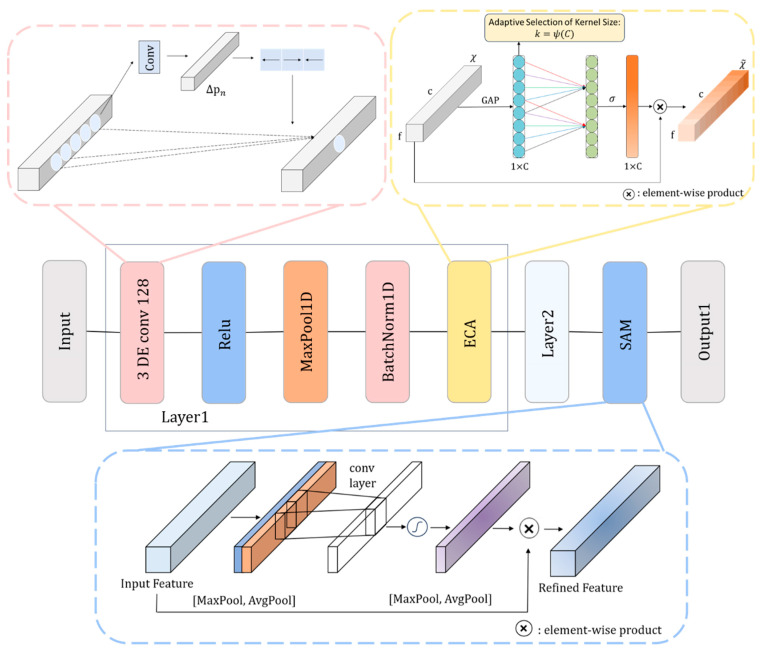
Spatial feature extraction module.

Figure 2 shows the detailed structure of the spatial feature extraction module. The module consists of two layers. The first layer (layer 1) includes a one-dimensional DFCNN (128 channels, with a convolution kernel size of 3), a ReLU activation function, a one-dimensional max-pooling layer (with a kernel size of 4), batch normalization, and ECA. The structure of the second layer (layer 2) is similar to the first, except that the number of DFCNN channels increases from 128 to 256. After two-layer feature extraction, the traffic data are input into the SAM layer and finally output as the spatial information feature map of the traffic data (output 1).

Network traffic has obvious one-dimensional characteristics. The process of a traditional one-dimensional convolution operation is as follows: Firstly, the input feature map is divided into multiple regions of the same size according to the size of the convolution kernel. In each region, the kernel’s weights are multiplied by the elements at the corresponding positions, and the results are summed to generate the corresponding output feature. To obtain a complete output feature map, the traditional one-dimensional convolution slides over the entire input feature map using a sliding window and performs the same operation at each position. Finally, the complete output feature graph is calculated. The convolution operation at any point $p_{0}$ on the input feature graph can be expressed by Formula (1).

(1) $$y\left(p_{0}\right)=\sum_{p_{n}\in R}w\left(p_{n}\right)*x(p_{0}+p_{n})$$

$p_{n}$ represents the offset of each point in the convolution kernel relative to the center point, that is, the current convolution kernel, which can be expressed as R={(−1,−1),(−1,0),…,(0,0),…,(1,0),(1,1)}. $w\left(p_{n}\right)$ represents the weight at the corresponding position of the convolution kernel. $x\left(p_{0}+p_{n}\right)$ represents the element value at position $p_{0}+p_{n}$ on the input feature graph. $y\left(p_{0}\right)$ represents the element value at position $p_{0}$ on the input feature graph.

In network intrusion detection tasks, intrusion behaviors often manifest as anomalies in local traffic. CNNs struggle to capture the full features of these local anomalies due to fixed sampling points. In contrast, a DFCNN adds a learnable offset $∆p_{n}$ to allow the convolution kernel to deform during operation, allowing for a more precise capture of the subtle features of intrusion behaviors. As shown in Figure 2, the offset $∆p_{n}$ is calculated by another convolution. In deformable convolution, it is necessary to increase the offset for each point based on Formula (1). As shown in Formula (2), the relative center offset is changed from the original offset $p_{n}$ to $p_{n}+∆p_{n}$.

(2) $$y\left(p_{0}\right)=\sum_{p_{n}\in R}w\left(p_{n}\right)*x(p_{0}+p_{n}+∆p_{n})$$

In the deformable convolution layer, we extract multiple channel features, with each channel regarded as a feature detector. However, this mechanism may overlook the key learning of important objects, potentially leading to overfitting. The ECA [33] at the end of each layer adaptively assigns different weights to each channel, focusing on the “importance” of features in the traffic, thus effectively extracting useful information.

Let the output of batch normalization be $\chi \in R^{f\times c}$, where f and c represent the flow features and the number of channels, respectively. As shown in the yellow box in Figure 2, in ECA, χ extracts aggregation features through global average pooling (GAP). Subsequently, ECA generates channel weights through a fast one-dimensional convolution (kernel size k), where the kernel size k is adaptively determined according to the channel dimension C. The channel weight is adjusted using the sigmoid activation function σ to scale the values within [0, 1]. A weight value of 0 indicates that the channel can be discarded, while a weight value of 1 signifies that the channel is fully preserved. Finally, the weighted feature $\tilde{\chi }$ is obtained by multiplying χ by the obtained weight matrix. This design captures important channel information, effectively alleviates overfitting, and improves the generalization ability of the network.

The channel attention mechanism emphasizes specific feature types, while the spatial attention mechanism locates the key positions in the feature map. To achieve comprehensive attention on “Which features are important?” (channel) and “Where is important?” (space), we use the spatial attention module (SAM) at the end of the spatial feature extraction module [34]. The SAM is located in the blue area of Figure 2. Firstly, the SAM performs average pooling and maximum pooling along the channel axis of the feature map to extract both global and local information. The pooled results are then concatenated to form a feature descriptor, effectively capturing key features of the feature map across different spatial locations. Next, the key spatial location information is captured through a convolution operation, and the spatial position information is passed through a sigmoid activation function to obtain weights in the range of [0, 1]. These weight values represent the importance of each spatial location; the closer the value is to 1, the more significant the features are at that location. Finally, the weights are assigned to the corresponding features. This design highlights important areas in the feature map, improving the network’s perception of key information.

### 3.3. Temporal Feature Extraction Module Based on Multi-Head Attention Mechanism

**Figure 3:**
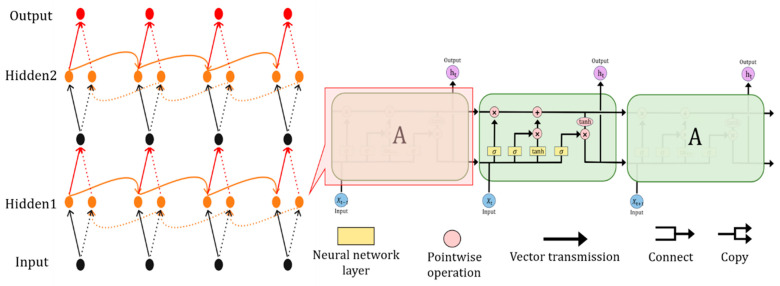
Overview of BiLSTM module.

The temporal feature extraction module consists of two layers: BiLSTM and MHA. The principle of BiLSTM is shown in Figure 3. BiLSTM is essentially a deep learning network that combines a bidirectional neural network with the LSTM structure. Building upon traditional LSTM, it captures richer context information by processing data in both the forward and backward directions simultaneously. The memory cell and various gates in LSTM are its core components. A memory cell solves the problem of long-term dependence in an RNN and dynamically selects the fate of information through the gating mechanism.

As shown in the green module in Figure 3, the input $X_{t}$ at the current time step sequentially passes through the forget gate, input gate, and output gate, ultimately producing the current time step’s output $h_{t}$. The output $f_{t}$ of the forgetting gate is multiplied by $C_{t-1}$ to control the retention of the previous time step’s memory, as shown in Formula (3). The input gate determines how much of the new input information $X_{t}$ at the current time step is written into the memory cell. The calculation process is as follows: Firstly, the weight $i_{t}$ of the input gate is computed using the σ activation function. Then, the candidate value $\tilde{C_{t}}$ is generated using the tanh function. Finally, $i_{t}$ is multiplied by $\tilde{C_{t}}$, as shown in Formulas (4) and (5). By combining the output $f_{t}$ of the forget gate and the output $i_{t}$ of the input gate, the memory unit $C_{t}$ at the current time step is updated, as shown in Formula (6). The output gate first calculates the output gate weight $o_{t}$ using the σ activation function. Then, $C_{t}$ is mapped onto an appropriate output range through the tanh function. Finally, the two values are multiplied to obtain the final output $h_{t}$ at the current time step, as shown in Formulas (7) and (8). This design enables LSTM to effectively preserve long-term dependencies while dynamically adjusting short-term memory, thus avoiding the vanishing gradient problem.

(3) $$f_{t}=\sigma (W_{f}\left[h_{t-1},X_{t}\right]+b_{f})$$

(4) $$i_{t}=\sigma (W_{i}\left[h_{t-1},X_{t}\right]+b_{i})$$

(5) $$\tilde{C_{t}}=tanh(W_{C}\left[h_{t-1},X_{t}\right]+b_{C})$$

(6) $$C_{t}=f_{t}*C_{t-1}+i_{t}*\tilde{C_{t}}$$

(7) $$o_{t}=\sigma (W_{o}\left[h_{t-1},X_{t}\right]+b_{o})$$

(8) $$h_{t}=o_{t}*tanh(C_{t})$$

BiLSTM processes sequential data in a step-by-step manner. Although the bidirectional mechanism enhances its ability to capture information from both directions, it still has a limited capacity for modeling long-range dependencies in long sequences. Intrusion behaviors in network traffic often exhibit such dependencies, and the multi-head attention (MHA) mechanism addresses this by concurrently attending to multiple, diverse dependencies. This allows it to capture a more comprehensive representation of attack behaviors, overcoming BiLSTM’s limitations in long-sequence modeling. Therefore, we incorporate MHA after the BiLSTM layer to capture inter-time step relationships and extract global context information. The location of the MHA mechanism is shown in Figure 1. By calculating the attention weight, the network can give different attention to the features of different time steps according to their importance so as to better focus on the critical time segments.

### 3.4. Equalization Loss v2

In this paper, the EQLv2 loss function [35] is used to balance the influence of each class at the algorithm level by assigning different weights to each class. The core calculation formula of the loss function can be expressed as weighted binary cross entropy (BCE), where the weighting factor depends on the class gradient, as shown in Formula (9):

(9) $$L_{i}=\frac{1}{N}\cdot \sum_{i=1}^{N}\left({CL}_{i}\cdot w_{i}\right)$$

By averaging the weighted cross-entropy loss of each category, the final EQLv2 loss value can be obtained, where N represents the number of classes, and ${CL}_{i}$ represents the cross-entropy loss of class i. The weighted cross-entropy loss is calculated by combining the weight $w_{i}$ of the i-th class. Formula (10) provides the calculation formula for the cross-entropy loss:

(10) $${CL}_{i}=-\left(t_{i}\cdot \log\left(\sigma \left(p_{i}\right)\right)+\left(1-t_{i}\right)\cdot \log\left(1-\sigma \left(p_{i}\right)\right)\right)$$

The formula calculates the loss for a positive sample ($t_{i}=1$) and a negative sample ($t_{i}=0$), respectively. If $t_{i}=1$, only $-\log\left(\sigma \left(p_{i}\right)\right)$ is valid, and in this case, the closer the model’s predicted probability is to 1. If $t_{i}=0$, $-\log\left(1-\sigma \left(p_{i}\right)\right)$ is valid. At this time, the closer the probability predicted by the model is to 0, the lower the loss value is. Here, $\sigma \left(x\right)$ represents the sigmoid function, which maps the prediction score $p_{i}$ of the i-th class onto a probability value between 0 and 1.

The working principle of EQLv2 is to calculate the corresponding weight by accumulating the ratio of positive to negative gradients of the classifier output during each backpropagation process. By dynamically adjusting the weight of positive and negative samples, it can inhibit the dominant role of common classes in model training. Formulas (11) and (12) define the calculation methods for the weights of positive and negative samples, respectively.

(11) $$w_{i}^{pos}=1+\alpha \cdot \left(1-w_{i}^{neg}\right)$$

(12) $$w_{i}^{neg}=\frac{1}{1+e^{-\gamma \cdot (r-\mu )}}$$

In Formula (11), α is a hyper-parameter, which is used to control the balance between the weights of positive and negative samples. The formula for calculating the weight of negative samples is given by (12). In this formula, γ and μ are hyperparameters that control the influence of gradient information on the weight of negative samples, while the parameter r represents the ratio between positive and negative samples. This ratio is obtained by accumulating the gradient ratio in the training task because the one-time gradient ratio cannot explain anything but will bring some noise to affect the normal learning of the network. Finally, the model parameter θ is updated through Formula (13).

(13) $$\theta =\theta -l\cdot \nabla_{\theta ℶ}$$

## 4. Experiment

### 4.1. Benchmark Dataset

The proposed ADFCNN-BiLSTM was evaluated on three standard datasets: NSL-KDD [36], UNSW-NB15 [37], and CIC-DDoS2019 [38]. The three datasets contain different types of attacks and numbers of features.

**Figure 4:**
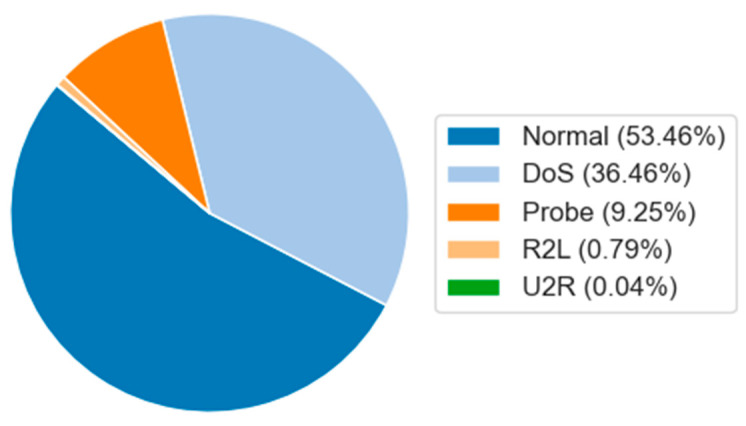
Data distribution of NSL-KDD dataset.

The NSL-KDD dataset is an improved version of the KDD’99 dataset. Redundancies and duplicate records have been removed, and the number of records in the training and test sets is complete, ensuring the comparability of evaluation results across different studies. Therefore, it is suitable as a benchmark dataset. The NSL-KDD dataset is generally divided into four attack types: DoS, Probe, R2L, and U2R. The data distribution is shown in Figure 4.

**Figure 5:**
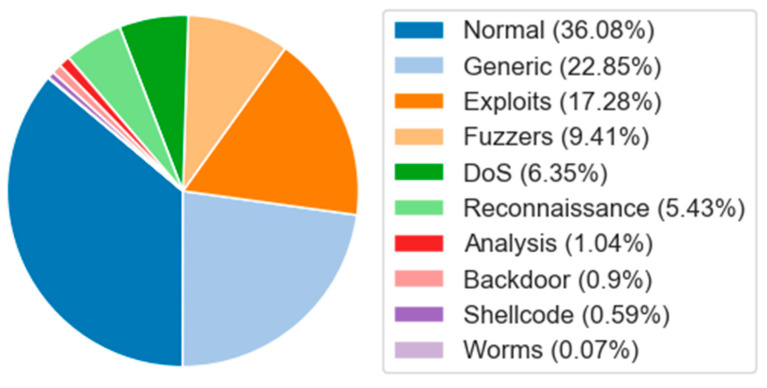
Data distribution of UNSW-NB15 dataset.

The raw network data of the UNSW-NB15 dataset is generated by the IXIA PerfectStorm tool from the Australian Network Security Center. This dataset contains both normal network traffic and various attack activities, comprehensively reflecting the modern network intrusion landscape. It includes nine types of attacks: fuzzers, analysis, backdoors, DoS, exploits, generic, reconciliation, shellcode, and worms. The data distribution is shown in Figure 5.

**Figure 6:**
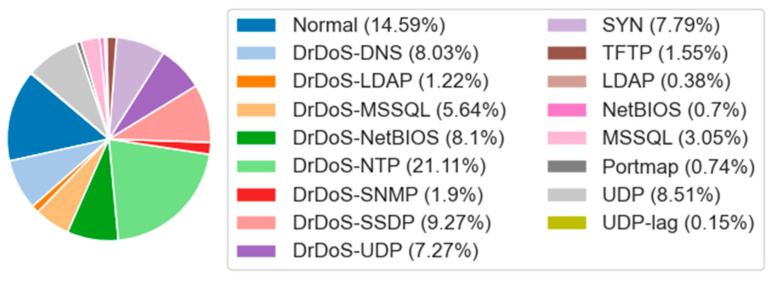
Data distribution of CIC-DDoS2019 dataset.

The CIC-DDoS2019 dataset provides an extended set of DDoS attacks, simulating modern network attacks. This dataset contains 14 attack types, namely, DrDoS DNS, DrDoS-LDAP, DrDoS-MSSQL, DrDoS-NetBIOS, DrDoS-NTP, DrDoS-SNMP, DrDoS-SSDP, DrDoS-UDP, SYN, TFTP, LDAP, NetBIOS, MSSQL, Portmap, and UDP Lag. The data distribution is shown in Figure 6. In our experiments, like other studies [24,39], we only selected a portion of the dataset (151,783 data points) for the experiment.

It can be seen from Figure 4, Figure 5 and Figure 6 that there is a significant data imbalance in the NSL-KDD, UNSW-NB15, and CIC-DDoS2019 datasets. In the NSL-KDD dataset, normal accounts for 54.22% of the entire dataset, while U2R only accounts for 0.18%. In the UNSW-NB15 dataset, normal accounts for 36.09%, while the minority classes, worms and shellcode, account for 0.07% and 0.59%, respectively. Similarly, in the CIC-DDoS2019 dataset, DrDoS-NTP accounts for 21.1%, while UDP-Lag only accounts for 0.1%.

### 4.2. Experimental Setup

The operating system used in this experiment was Ubuntu 18.04, equipped with 16GB of GPU memory. The CUDA version was 11.8, and the GPU model was the NVIDIA Tesla T4. The proposed network was implemented in a Python 3.10 environment, used the PyTorch 2.1.2 deep learning framework, and employed the Adam optimizer for training. The training process was set for 50 epochs. For the NSL-KDD, UNSW-NB15, and CIC-DDoS2019 datasets, the learning rates were set to 0.0005, 0.0005, and 0.001, respectively.

### 4.3. Evaluation Metrics

The performance and robustness of the proposed network were tested using three evaluation indicators: accuracy, detection rate, and false-positive rate. True positives (TPs) and true negatives (TNs) indicate the proportion of network traffic correctly classified as normal and abnormal, respectively. The misclassified normal and abnormal network traffic can be represented as false positives (FPs) and false negatives (FNs). The specific descriptions are as follows:

(1) Accuracy

The accuracy refers to the proportion of all samples correctly classified by the detection system, but it may be affected by imbalanced data. The calculation method is shown in Formula (14).

(14) $$Acc=\frac{TN+TP}{TN+FP+FN+TP}$$

(2) Detection rate

The detection rate, also called recall, measures the proportion of all real intrusion samples that are correctly identified, reflecting the model’s ability to detect intrusions. The calculation method is shown in Formula (15).

(15) $$DR=\frac{TP}{TP+FN}$$

(3) False-positive rate

The false-positive rate refers to the proportion of normal samples incorrectly identified as intrusions, reflecting the model’s false alarm rate. The calculation method is shown in Formula (16).

(16) $$FPR=\frac{FP}{FP+TN}$$

### 4.4. Experimental Evaluation

In the entire experiment evaluation, we used both multi-class and binary classification to train the ADFCNN-BiLSTM network.

#### 4.4.1. Comparative Experiment

We compared the results of the proposed ADFCNN-BiLSTM with those of other state-of-the-art networks. From the data in Table 1, we can see that ADFCNN-BiLSTM performed well on the NSL-KDD dataset, with an accuracy rate of 99.17%, a detection rate of 99.98%, and a false alarm rate of 0.01%, which were significantly better than those of the other compared models. In contrast, XGBoost had a slightly higher accuracy rate of 99.64%, but its detection rate was only 98.12%, and its false alarm rate was 0.134%, which was obviously inferior. Although the detection rate of the IDS-INT model reached 99.00%, its accuracy rate was 98.45%, and its false alarm rate was also high. In addition, the overall performance of GBDT was the worst, with only 81.80% accuracy and a 67.20% detection rate, while its false alarm rate was as high as 3.80%. Compared with the latest methods, ADFCNN-BiLSTM led in overall performance.

Table 2 shows the performance comparison of different networks on the UNSW-NB15 dataset. GBDT [40] performed the worst in all the indicators. For TACGAN [18], ENIDS [23], NAEF [42], PIO-DT [48], and K-GetNID [43], all indicators were inferior to those of ADFCNN-BiLSTM. Although the accuracy of TMG-GAN [49] was as high as 99.70%, its detection rate was 95.63%, and its false alarm rate was 0.02%, its comprehensive performance was still higher than that of the proposed network. DE-VIT [27] was close to the proposed network, with an accuracy rate of 97.20%, a detection rate of 94.89%, and a false alarm rate of 1.40%, but there were still gaps. The accuracy rate of GA-RF [50] was 92.80%, lower than that of the proposed network. In conclusion, the performance of the proposed network on the UNSW-NB15 dataset was better than that of the other compared networks.

According to the results on the CIC-DDoS2019 dataset in Table 3, ADFCNN-BiLSTM was close to the best in both the detection rate and false alarm rate and was far better than the other methods, such as KS-DDoS and optimized LSTM, demonstrating the advantage of lower false alarms. In terms of accuracy, ADFCNN-BiLSTM reached 93.81%, which was slightly lower than optimized LSTM but superior to KS-DDoS and random forest. To sum up, ADFCNN-BiLSTM strikes a good balance between its detection rate and false alarm rate, with its overall performance approaching optimal levels, making it especially suitable for the efficient and accurate detection of DDoS attacks.

Table 4 lists the detection rates of the following detection networks—CNN-BiLSTM-Attention [24], CANET [39], CNN-BiLSTM [13], and ADFCNN-BiLSTM—for each class of the UNSW-NB15 dataset. ADFCNN-BiLSTM performed well in detecting normal, fuzzer, DoS, reconciliation, analysis, backdoor, shellcode, and worm attacks. The detection rates for shellcode and worms even reached 100%, demonstrating the advantages of our model in detecting abnormal traffic with small sample sizes.

**Figure 7:**
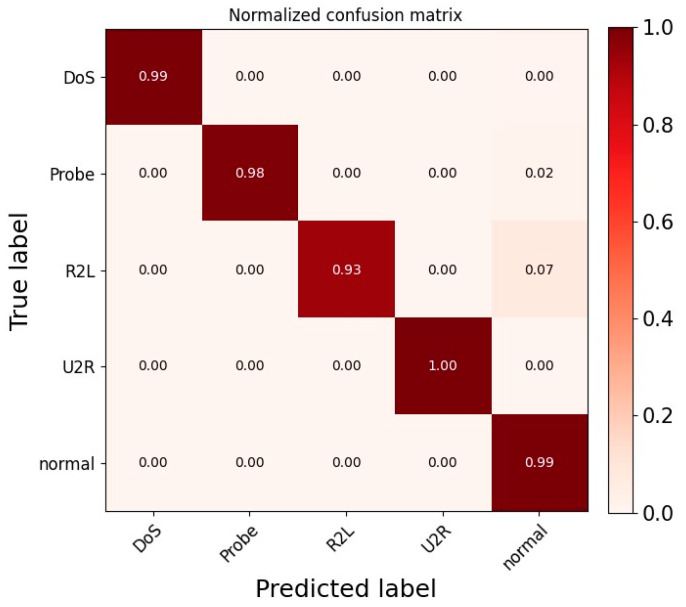
Confusion matrix using NSL-KDD dataset.

**Figure 8:**
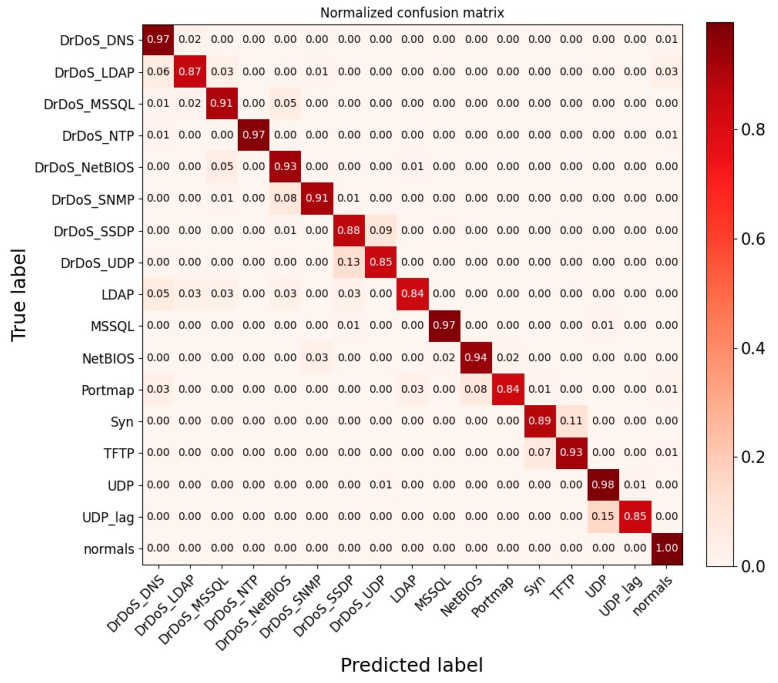
Confusion matrix using CIC-DDoS2019 dataset.

In addition, we also evaluated the multi-class classification performance of the ADFCNN-BiLSTM network on the NSL-KDD and CIC-DDoS2019 datasets. Figure 7 shows a confusion matrix that illustrates the detection performance of the proposed network for different classes of attacks on the NSL-KDD dataset. ADFCNN-BiLSTM achieved detection rates above 0.99 for the DoS, U2R, and normal classes. When the true label was probed, the prediction probability was 0.98, while the prediction probability for R2L was 0.93. Of these two classes, 0.02 and 0.07 were misclassified as normal traffic, respectively. This is because the normal class has more samples than probe and R2L, making it easier for the network to learn the underlying characteristics of normal traffic. In general, ADFCNN-BiLSTM performed well across most classes, indicating that the network can effectively address the class imbalance problem in the NSL-KDD dataset. Figure 8 shows the confusion matrix for the CIC-DDoS2019 dataset.

On the whole, ADFCNN-BiLSTM achieved good multi-class classification results on the NSL-KDD, UNSW-NB15, and CIC-DDoS2019 datasets, demonstrating that the network can effectively address the imbalance problem in network traffic data and possesses strong robustness and generalization ability.

#### 4.4.2. K-Fold Cross-Validation

In order to evaluate the stability and generalization ability of the ADFCNN-BiLSTM network for intrusion traffic, this paper adopted the K-fold cross-validation method. The multi-class and binary classification performance of the network was evaluated on the NSL-KDD, UNSW-NB15, and CIC-DDoS2019 datasets. The value of K ranged from 2 to 10.

Table 5 shows the K-fold cross-validation results of the network on the NSL-KDD dataset. In the table, we can observe that the classification accuracy fluctuated between 98.61% and 98.90%, ranging from 20% to 10%, with an average accuracy of 98.83%. The detection rate was around 99.98%, and the false positive rate was about 0.01%. This shows that the performance of the model in multi-classification tasks was excellent and stable. The average accuracy of binary classification was 98.94%, with the highest being 99.03%, indicating that the network also had high accuracy in the binary classification task. In general, the K-fold cross-validation results of different fold numbers were relatively stable. The accuracy and detection rates were very high, and the false alarm rate was very low, whether in multi-classification or binary classification tasks. These results demonstrate that the ADFCNN-BiLSTM network has a strong classification ability and reliability.

According to the results in Table 6, the proposed network performed well on the UNSW-NB15 dataset. The average multi-classification accuracy (Multi-Acc) was 95.07%, with a fluctuation range of 94.43% to 96.03%. It was stable but slightly lower than on the NSL-KDD dataset. The average binary classification accuracy (Binary Acc) was 96.68%, with the highest being 97.28% (in the fourth fold), showing high accuracy. The average detection rate (DR) was 97.23%, indicating that the network performed well in identifying attack samples. The average false-positive rate (FPR) was only 0.01%, remaining at a very low level, reflecting the network’s reliability. In general, the network performed slightly worse on the UNSW-NB15 dataset than on the NSL-KDD dataset, but its accuracy, detection rate, and low false alarm rate demonstrated a good generalization ability and practical application value.

The multi-classification and binary classification results for the CIC-DDoS2019 dataset are shown in Table 7. The average accuracy of binary classification was 99.89%, demonstrating a high classification accuracy. The average multi-classification accuracy was 89.94%, which was lower than that of binary classification but still performed well. The average detection rate was 91.65%, indicating that the model was highly efficient in identifying attacks. The detection rate of the 10th fold was 91.90%, the highest value, and the false-positive rate was only 0.01%, the lowest value, showing that the classification performance of this fold was the most balanced. In general, the network performed stably on the CIC-DDoS2019 dataset, with binary classification performing better than multi-classification, which proves the effectiveness of the model in DDoS attack detection.

#### 4.4.3. Ablation Experiment

We conducted ablation experiments on the UNSW-NB15 dataset to evaluate the contribution of various components in ADFCNN-BiLSTM. The experimental results are shown in Table 8. The baseline refers to our proposed ADFCNN-BiLSTM. ‘Baseline without Class Balancing’ refers to the network after removing the data-balancing algorithms, SMOTE and ENN. ‘Baseline without ADFCNN’ is a network using traditional convolution. ‘Baseline without ECA’ represents the removal of the channel attention mechanism. ‘Baseline without SAM’ refers to the removal of the spatial attention mechanism, and ‘Baseline without MHA’ refers to the removal of the multi-head attention mechanism.

It can be seen from Table 8 that the accuracy rate of the baseline network reached 98.70%, the detection rate was 99.74%, and the false alarm rate was 0%, showing excellent performance. After removing class balancing, the accuracy rate dropped slightly to 97.99%, but the detection rate and false alarm rate showed almost no change, indicating that class balancing had a limited impact on the network performance. After removing the DFCNN, the accuracy rate dropped to 97.45%, the detection rate slightly increased to 99.93%, and the false alarm rate increased to 0.01%, indicating that the DFCNN played an important role in improving the accuracy of the network. After removing ECA, SAM, and MHA, the accuracy rate dropped to 97.44%, 97.33%, and 97.41%, respectively, showing that the removal of each component resulted in a slight decrease in accuracy. The detection rate dropped slightly, but the false alarm rate remained at 0% or 0.01%, indicating that these components positively impacted the accuracy of the model but had minimal effect on the detection rate and false alarm rate. In general, each component of the baseline network positively contributed to the network performance.

In order to verify the effectiveness of the EQLv2 loss function in solving the data imbalance problem, we conducted comparative experiments on the UNSW-NB15 dataset for ADFCNN-BiLSTM using EQLv2 and cross-entropy loss (CE), as shown in Table 9. The results show that the detection rate of EQLv2 was significantly higher than that of CE for minority categories such as exploits and analysis. The detection rate of the generic and fuzzy categories with CE was slightly higher than with EQLv2, but the difference was small. For categories like normal and worms, both CE and EQLv2 achieved high detection rates. For the DoS category, both loss functions yielded relatively low detection rates. Overall, compared with CE, the EQLv2 loss function had obvious advantages in addressing data imbalance. Therefore, ADFCNN-BiLSTM using the EQLv2 loss function can effectively solve the data imbalance problem.

## 5. Conclusions

In this paper, we propose the ADFCNN-BiLSTM network. ADFCNN-BiLSTM combines SMOTE, ENN resampling techniques, and the EQLv2 loss function to mitigate the impact of data imbalance during network training. We use a DFCNN to extract spatial features, while ECA and SAM are employed to emphasize the importance of key features for classification. BiLSTM is used to extract temporal features, and MHA is applied after BiLSTM to identify key temporal features.

We used three standard datasets to evaluate the proposed ADFCNN-BiLSTM. The experimental results show that the proposed network leads in NIDS tasks. Additionally, we conducted cross-validation experiments to demonstrate that ADFCNN-BiLSTM has a good generalization ability. Finally, we performed ablation experiments to validate the rationality of each component of ADFCNN-BiLSTM.

However, during the experiments, we found the limitations of the SMOTE and ENN resampling methods due to their lack of adaptability. Therefore, future studies may involve exploring the automation of calculating the number of samples for each class in resampling techniques to identify the optimal resampling targets and maximize the mitigation of data imbalance at the data level. In addition, since ADFCNN-BiLSTM is a supervised learning model, it struggles to differentiate between legitimate traffic and new intrusions when encountering previously unseen traffic. To overcome this, we will employ the transfer learning technique to assess how well they function on unknown network traffic. Moreover, integrating advanced technologies such as transformers and large language models could further enhance the detection performance.

## Figures and Tables

**Table 1:** Network performance comparison for NSL-KDD dataset.

Network	Acc (%)	DR (%)	FPR (%)
Ours	99.17	99.98	0.01
GBDT [40]	81.80	67.20	3.80
XGBoost [41]	99.64	98.12	0.134
NAEF [42]	87.80	88.20	1.23
SCDAE-CNN-BiLSTM-Attention [43]	93.26	94.26	N/A
IDS-INT [22]	98.45	99.00	0.93
Dbde-qda [44]	97.40	97.40	3.00
CFS-BA [45]	87.30	87.40	3.19
LightGBM [46]	98.20	98.20	1.70
GWO-PSO-RF [47]	99.20	N/A	N/A

**Table 2:** Network performance comparison for UNSW-NB15 dataset.

Network	Acc (%)	DR (%)	FPR (%)
Ours	98.70	99.74	0.01
GBDT [40]	71.70	62.90	26.90
TACGAN [18]	92.39	94.03	7.80
ENIDS [23]	90.60	90.50	0.03
NAEF [42]	85.7	87.3	0.02
TMG-GAN [49]	99.70	95.63	0.02
DE-VIT [27]	97.20	94.89	1.40
K-GetNID [43]	86.12	86.32	0.70
PIO-DT [48]	91.70	89.40	3.40
GA-RF [50]	92.80	N/A	3.3

**Table 3:** Network performance comparison for CIC-DDoS2019 dataset.

Network	Acc (%)	DR (%)	FPR (%)
Ours	93.81	99.65	0.06
SCDAE-CNN-BiLSTM-Attention [43]	93.26	88.23	N/A
KS-DDoS [51]	91.23	92.00	4.27
Optimized LSTM [52]	94.01	85.02	3.74
Random forest [53]	89.05	89.00	2.89

**Table 4:** Comparison of detection rate for each class.

Class	Ours	CNN-BiLSTM-Attention [24]	CANET [39]	CNN-BiLSTM [13]
Normal	99%	99%	99%	95%
Generic	91%	99%	99%	98%
Exploits	44%	92%	85%	92%
Fuzzers	85%	85%	84%	54%
DoS	50%	27%	46%	5%
Reconnaissance	90%	85%	84%	73%
Analysis	83%	25%	21%	6%
Backdoor	62%	20%	14%	7%
Shellcode	100%	90%	87%	0%
Worms	100%	61%	89%	100%

**Table 5:** Multi-classification and binary-classification results on NSL-KDD dataset.

Fold	Multi-Acc (%)	Binary Acc (%)	DR (%)	FPR (%)
2	98.85	98.96	99.98	0.01
4	98.88	98.98	99.99	0.00
6	98.61	98.75	99.97	0.01
8	98.89	98.98	99.97	0.01
10	98.90	99.03	99.99	0.01
Average	98.83	98.94	99.98	0.01

**Table 6:** Multi-classification and Binary-classification results on UNSW-NB15 datasets.

Fold	Multi-Acc(%)	Binary Acc(%)	DR(%)	FPR(%)
2	96.03	97.14	98.06	0.01
4	95.28	97.28	97.35	0.01
6	94.76	96.86	97.04	0.01
8	94.86	96.81	97.05	0.01
10	94.43	95.32	96.65	0.01
Average	95.07	96.68	97.23	0.01

**Table 7:** Multi-classification and binary-classification results on CIC-DDoS2019 dataset.

Fold	Multi-Acc (%)	Binary Acc (%)	DR (%)	FPR (%)
2	89.67	99.89	91.15	0.05
4	90.82	99.91	92.54	0.07
6	88.64	99.88	90.55	0.07
8	90.55	99.89	92.11	0.05
10	90.01	99.88	91.90	0.01
Average	89.94	99.89	91.65	0.05

**Table 8:** Results of ablation experiments on the UNSW-NB15 dataset.

Model	Acc (%)	DR (%)	FPR (%)
Baseline	98.70	99.74	0.00
Baseline without Class Balancing	97.99	99.73	0.00
Baseline without DFCNN	97.45	99.93	0.01
Baseline without ECA	97.44	99.92	0.00
Baseline without SAM	97.33	99.87	0.00
Baseline without MHA	97.41	99.91	0.00

**Table 9:** Comparison of loss function.

Class	EQL v2	CE
Normal	99%	100%
Generic	91%	96%
Exploits	44%	14%
Fuzzers	85%	90%
DoS	50%	43%
Reconnaissance	90%	65%
Analysis	83%	0%
Backdoor	62%	38%
Shellcode	100%	86%
Worms	100%	100%

## Data Availability

This study used the NSL-KDD, USNW-NB15, and CICDDoS2019 datasets, all of which are publicly available datasets. They can be found at https://www.unb.ca/cic/datasets/nsl.html, https://research.unsw.edu.au/projects/unsw-nb15-dataset, and https://www.unb.ca/cic/datasets/ddos-2019.html (all accessed on 23 January 2025).

## References

[1] Gao M., Ma L., Liu H., Zhang Z., Ning Z., Xu J. (2020). Malicious network traffic detection based on deep neural networks and association analysis. Sensors.

[2] Elhanashi A., Dini P., Saponara S., Zheng Q. (2023). Integration of Deep Learning into the IoT: A Survey of Techniques and Challenges for Real-World Applications. Electronics.

[3] Javadpour A., Ja’fari F., Taleb T., Shojafar M., Benzaïd C. (2024). A comprehensive survey on cyber deception techniques to improve honeypot performance. Comput. Secur..

[4] Ahmad Z., Shahid Khan A., Wai Shiang C., Abdullah J., Ahmad F. (2021). Network intrusion detection system: A systematic study of machine learning and deep learning approaches. Trans. Emerg. Telecommun. Technol..

[5] Asad H., Adhikari S., Gashi I. (2024). A perspective–retrospective analysis of diversity in signature-based open-source network intrusion detection systems. Int. J. Inf. Secur..

[6] Yang Z., Liu X., Li T., Wu D., Wang J., Zhao Y., Han H. (2022). A systematic literature review of methods and datasets for anomaly-based network intrusion detection. Comput. Secur..

[7] Padmaja R., Challagundla P.R. Exploring a two-phase deep learning framework for network intrusion detection. Proceedings of the 2024 IEEE International Students’ Conference on Electrical, Electronics and Computer Science (SCEECS).

[8] Disney D.A., Yugha R., Karachi S.B., Gangadevi E., Balusamy B., Gite S. (2024). An AI-driven based cybersecurity system for network intrusion detection system in hybrid with EPO and CNNet-LAM. Proceedings of the 2024 IEEE International Conference on Computing, Power and Communication Technologies (IC2PCT).

[9] Parameswari A., Ganeshan R., Ragavi V., Shereesha M. (2024). Hybrid rat swarm hunter prey optimization trained deep learning for network intrusion detection using CNN features. Comput. Secur..

[10] Azarudeen K., Ghulam D., Rakesh G., Sathaiah B., Vishal R. (2024). Intrusion detection system using machine learning by RNN method. Proceedings of the E3S Web of Conferences.

[11] Wang F., Dong Z. (2024). Fusion of spiral convolution-LSTM for intrusion detection modeling. Comput. Mater. Contin..

[12] Aljehane N.O., Mengash H.A., Eltahir M.M., Alotaibi F.A., Aljameel S.S., Yafoz A., Alsini R., Assiri M. (2024). Golden jackal optimization algorithm with deep learning assisted intrusion detection system for network security. Alex. Eng. J..

[13] Sinha J., Manollas M. (2020). Efficient deep CNN-BiLSTM model for network intrusion detection. Proceedings of the 2020 3rd International Conference on Artificial Intelligence and Pattern Recognition (AIPR ’20).

[14] Zhang Y.D., Chen S.Y., Peng Y.H., Yang J. (2019). Survey of deep learning-based network intrusion detection. J. Guangzhou Univ. (Nat. Sci. Ed.).

[15] Puri A., Gupta M.K. (2019). Comparative analysis of resampling techniques under noisy imbalanced datasets. Proceedings of the 2019 International Conference on Issues and Challenges in Intelligent Computing Techniques (ICICT).

[16] Zhu Y., Yan Y., Zhang Y., Zhang Y. (2020). EHSO: Evolutionary hybrid sampling in overlapping scenarios for imbalanced learning. Neurocomputing.

[17] Xw A., Jian X.A., Tz B., Lj A. (2021). Local distribution-based adaptive minority oversampling for imbalanced data classification. Neurocomputing.

[18] Ding H., Chen L., Dong L., Fu Z., Cui X. (2022). Imbalanced data classification: A KNN and generative adversarial networks-based hybrid approach for intrusion detection. Future Gener. Comput. Syst..

[19] Li Z., Huang C., Qiu W. (2024). An intrusion detection method combining variational auto-encoder and generative adversarial networks. Comput. Netw..

[20] Chawla N.V., Bowyer K.W., Hall L.O., Kegelmeyer W.P. (2002). SMOTE: Synthetic minority over-sampling technique. J. Artif. Intell. Res..

[21] Xu Z., Shen D., Nie T., Kou Y. (2020). A hybrid sampling algorithm combining m-SMOTE and ENN based on random forest for medical imbalanced data. J. Biomed. Inform..

[22] Ullah F., Ullah S., Srivastava G., Lin J.C.W. (2024). IDS-INT: Intrusion detection system using transformer-based transfer learning for imbalanced network traffic. Digit. Commun. Netw..

[23] Sayem I.M., Sayed M.I., Saha S., Haque A. (2024). ENIDS: A deep learning-based ensemble framework for network intrusion detection systems. IEEE Trans. Netw. Serv. Manag..

[24] Shin Y., Kim M., Kim H. (2024). Towards unbalanced multiclass intrusion detection with hybrid sampling methods and ensemble classification. Appl. Soft Comput..

[25] Zhou Y., Mazzuchi T.A., Sarkani S. (2020). M-AdaBoost-A based ensemble system for network intrusion detection. Expert Syst. Appl..

[26] Xu B., Sun L., Mao X., Liu C., Ding Z. (2024). Strengthening network security: Deep learning models for intrusion detection with optimized feature subset and effective imbalance handling. Comput. Mater. Contin..

[27] Dai W., Li X., Ji W., He S. (2024). Network intrusion detection method based on CNN, BiLSTM, and attention mechanism. IEEE Access.

[28] Nguyet L., Hai T.H. Evaluation of deep CNN-BiLSTM model on diverse datasets. Proceedings of the 2023 International Conference on Advanced Computing and Analytics (ACOMPA).

[29] Gao J. (2022). Network intrusion detection method combining CNN and BiLSTM in cloud computing environment. Comput. Intell. Neurosci..

[30] Zhang Y., Guo Z., Ma H., Guan Q., Jiang W., Li W. (2024). An efficient CNN+ Sparse Transformer-based intrusion detection method for IoT. Advanced Intelligent Computing Technology and Applications.

[31] Jouhari M., Guizani M. (2024). Lightweight CNN-BiLSTM based intrusion detection systems for resource-constrained IoT devices. arXiv.

[32] Said R.B., Sabir Z., Askerzade I. (2023). CNN-BiLSTM: A hybrid deep learning approach for network intrusion detection system in software-defined networking with hybrid feature selection. IEEE Access.

[33] Wang Q., Wu B., Zhu P., Li P., Zuo W., Hu Q. ECA-Net: Efficient channel attention for deep convolutional neural networks. Proceedings of the IEEE/CVF Conference on Computer Vision and Pattern Recognition.

[34] Woo S., Park J., Lee J.Y., Kweon I.S. Cbam: Convolutional block attention module. Proceedings of the European Conference on Computer Vision (ECCV).

[35] Tan J., Lu X., Zhang G., Yin C., Li Q. Equalization loss v2: A new gradient balance approach for long-tailed object detection. Proceedings of the IEEE/CVF Conference on Computer Vision and Pattern Recognition (CVPR).

[36] Meena G., Choudhary R.R. A review paper on IDS classification using KDD 99 and NSL KDD dataset in WEKA. Proceedings of the 2017 International Conference on Computer, Communications and Electronics (Comptelix).

[37] Moustafa N., Slay J. UNSW-NB15: A comprehensive data set for network intrusion detection systems (UNSW-NB15 network data set). Proceedings of the 2015 Military Communications and Information Systems Conference (MilCIS).

[38] Sharafaldin I., Lashkari A.H., Hakak S., Ghorbani A.A. Developing realistic distributed denial of service (DDoS) attack dataset and taxonomy. Proceedings of the 2019 International Carnahan Conference on Security Technology (ICCST).

[39] Ren K., Yuan S., Zhang C., Shi Y., Huang Z. (2023). CANET: A hierarchical cnn-attention model for network intrusion detection. Comput. Commun..

[40] Ren H., Tang Y., Dong W., Ren S., Jiang L. (2023). DUEN: Dynamic ensemble handling class imbalance in network intrusion detection. Expert Syst. Appl..

[41] Widodo A.O., Setiawan B., Indraswari R. (2024). Machine learning-based intrusion detection on multi-class imbalanced dataset using SMOTE. Procedia Comput. Sci..

[42] Yu X., Lu Y., Jiang F., Hu Q., Du J., Gong D. (2024). A cross-domain intrusion detection method based on nonlinear augmented explicit features. IEEE Trans. Netw. Serv. Manag..

[43] Wang M., Yang N., Weng N. (2024). K-GetNID: Knowledge-guided graphs for early and transferable network intrusion detection. IEEE Trans. Inf. Forensics Secur..

[44] Zorarpaci E. (2024). A fast intrusion detection system based on swift wrapper feature selection and speedy ensemble classifier. Eng. Appl. Artif. Intell..

[45] Zhou Y., Cheng G., Jiang S., Dai M. (2020). Building an efficient intrusion detection system based on feature selection and ensemble classifier. Comput. Network..

[46] Liu J., Gao Y., Hu F. (2021). A fast network intrusion detection system using adaptive synthetic oversampling and LightGBM. Comput. Secur..

[47] Keserwani P.K., Govil M.C., Pilli E.S., Govil P. (2021). A smart anomaly-based intrusion detection system for the Internet of Things (IoT) network using GWO–PSO–RF model. J. Reliab. Intell. Environ..

[48] Alazzam H., Sharieh A., Sabri K.E. (2020). A feature selection algorithm for intrusion detection system based on pigeon inspired optimizer. Expert Syst. Appl..

[49] Ding H., Sun Y., Huang N., Shen Z., Cui X. (2023). TMG-GAN: Generative adversarial networks-based imbalanced learning for network intrusion detection. IEEE Trans. Inf. Forensics Secur..

[50] Ren J., Guo J., Qian W., Yuan H., Hao X., Jingjing H. (2019). Building an effective intrusion detection system by using hybrid data optimization based on machine learning algorithms. Secur. Commun. Netw..

[51] Patil N.V., Krishna C.R., Kumar K. (2022). KS-DDoS: Kafka streams-based classification approach for DDoS attacks. J. Supercomput..

[52] Packialatha A. (2023). Hybrid classification model with tuned weight for cyber-attack detection: Big data perspective. Adv. Eng. Softw..

[53] Patil N.V., Krishna C.R., Kumar K. (2022). SSK-DDoS: Distributed stream processing framework based classification system for DDoS attacks. Clust. Comput..

